# Establishment of a laboratory colony of *Pressatia choti* (Diptera: Psychodidae), a suspected vector of *Leishmania braziliensis*

**DOI:** 10.1590/0037-8682-0060-2024

**Published:** 2024-06-10

**Authors:** Joanna Alexandre, Débora Elienai de Oliveira Miranda, Fernando José da Silva, Filipe Dantas-Torres, Sinval Pinto Brandão-Filho

**Affiliations:** 1 Instituto Aggeu Magalhães, Fundação Oswaldo Cruz, Departamento de Imunologia, Recife, PE, Brasil.

**Keywords:** Pressatia choti, Life cycle, *Lutzomyia* spp

## Abstract

**Background:**

*Pressatia choti* is a common sand fly found in the Atlantic Forest of Brazil, which is suspected to be involved in the transmission of *Leishmania braziliensis*. Herein, we aimed to establish a *Pr*. *choti* laboratory colony.

**Methods:**

Wild-caught female sand flies were blood fed on hamsters and maintained under controlled conditions (temperature: 26 °C; relative humidity: 70%).

**Results:**

Of the 301 collected female sandflies, 288 were identified as *Pr. choti*. The life cycle duration ranged from 31 to 56 days.

**Conclusions:**

We successfully established a *Pr. choti* colony, whose biological parameters were similar to those of other neotropical sand flies.

*Pressatia choti* (Floch and Abonnenc, 1941) is a common phlebotomine sandfly species found in the remaining areas of the Atlantic Forest biome in Brazil^1^. In Pernambuco, *Pr. choti* is abundant in some municipalities, including areas where cutaneous leishmaniasis caused by *Leishmania* (*Viannia*) *braziliensis* is endemic^2^ ^,^ ^3^. In a study conducted in the Atlantic Forest region of Pernambuco^4^, *Pr. choti* females were willing to feed on humans. Recently, molecular evidence of *L.* (*V.*) *braziliensis* DNA in *Pr*. *choti* females were observed in military training camp^2^ ^,^ ^5^. This indicates that *Pr. choti* could be a possible vector for *L*. (*V*.) *braziliensis*, although further research is needed to confirm its vector competence and capacity through experimental studies of infection under laboratory conditions.

Demonstrating the development of *Leishmania* parasites in female sand flies under experimental conditions is fundamental for studying their vector competence. Therefore, the establishment of laboratory colonies of phlebotomine sand flies is pivotal for vector competence studies. However, this has been a critical and limiting factor because of the difficulties in establishing and maintaining colonies of these insects^6^. In 2017, according to Lawyer et al.^7^, there were 21 phlebotomine sand fly species colonized in 35 laboratories distributed across 18 countries. In Brazil, laboratory colonies of three species, *Lutzomyia longipalpis, Migonemyia migonei*, and *Nyssomyia neivai have been* established^7^.

During the process of establishing a laboratory colony of a given phlebotomine sand fly species, identifying its preferred blood source, finding the ideal conditions for temperature and relative humidity, and limiting the growth of fungi during the larval phase are some critical points^8^. Some phlebotomine sand flies are easy to maintain under laboratory conditions, such as *Lu. longipalpis* and *Phlebotomus papatasi* ^6^. However, other species are difficult to colonize and have low productivity when kept in under laboratory conditions, like *Ny. intermedia* and *Ny. neivai* ^9^. Despite these difficulties, the establishment of phlebotomine sand fly colonies is crucial for studying the parasite-vector relationships and methods for controlling these insects^7^ ^,^ ^10^. Therefore, considering the potential roles of *Pr. choti* as a vector, the objective of this study was to establish and maintain a colony of this phlebotomine sand fly species under laboratory conditions.

The captures of the phlebotomine sand fly specimens were carried out at a military training camp (Campo de Instrução Militar Marechal Newton Cavalcanti - CIMNIC), located in the Atlantic Forest region of Pernambuco. Ten light traps (CDC model) were installed at preselected sites (according to a previous study)^2^, from 5:00 pm to 6:00 am, on four consecutive nights in May 2019. The captured specimens were transferred to cages (20 cm × 20 cm) using manual aspirators and wrapped in a plastic bag containing cotton soaked in distilled water to maintain humidity. The cages were packed in boxes, which also had cotton with distilled water and transported to the insectary of Aggeu Magalhães Institute/Fiocruz in Recife, Pernambuco. In the laboratory, the captured phlebotomine sand flies were blood-fed (animal ethics committee approval: 092/2015-2020) for 1 h on a hamster (*Mesocricetus auratus*) that was previously anesthetized intramuscularly with ketamine (0.1 mL/100 g). After 24 h, 327 fed females were transferred individually to acrylic tubes (RN Embalagen^®^, 10 mL) containing filter paper, then packed in a plastic box with autoclaved sand that was moistened with water to maintain humidity. After death, the females (head and last two abdominal segments) were slide-mounted in Berlese solution and identified morphologically^11^. The structures used for identification were the palpi, antennae, ascoids, pharynx, cibarium, cibarial arch, pigmented area, labial fork (suture), spermathecae, and spermathecal ducts. Considering the morphological similarities between the females of *Pressatia* spp., *Pr. choti* was identified through its association with the males of this species, which was the only species present in the study area^3^.

In total, 301 female phlebotomine sand flies were identified (288 *Pr. choti*, seven *Psychodopygus complexus/wellcomei*, two *Evandromyia evandroi*, two *Trichopygomyia longispina*, one *Nyssomyia umbratilis*, and one *Nyssomyia yulli pajoti*) with *Pr. choti* representing ~88% of the specimens. Twenty-six females could not be identified because the structures (e.g., spermathecae) necessary for morphological identification were not visible.

After identification, eggs of the same species were transferred to larger pots for continued development. The establishment and maintenance of *Pr. choti* colonies was carried out according to the method described by Volf and Volfova^6^. After oviposition, the eggs were transferred with the aid of a paintbrush to plastic pots (Nalgene^®^, 500 mL) with a plaster layer in the bottom. After hatching, the larvae were fed a mixture of rabbit feces and rabbit food in a 1:1 ratio, and the pots containing the larvae were kept in plastic boxes containing autoclaved, moist sand to maintain humidity. The insects were exposed to a photoperiod of 14 h of light and 10 h of darkness and maintained at a temperature of 26 ºC and 70% relative humidity, with daily checks for the presence of fungi and mites. The breeding containers were inspected daily for life-cycle assessments. The adult sand flies were kept in breeding cages (20 × 20 cm) wrapped in a transparent plastic bag containing cotton soaked in distilled water and maintained with sugar solution for 3-4 days, after which they were subjected to blood feeding. The sugar solution was removed 48 h prior to blood feeding. These results were calculated based on the data obtained from eight generations.

No special issues were observed during blood feeding and 90-100% of the females were successfully fed on hamsters. The other food sources commonly used for feeding phlebotomine sand flies, such as mice and rabbits^6^ ^,^ ^12^ ^,^ ^13^, were not tested. The complete life cycle from egg to adult emergence ranged from 31 to 56 days. The egg incubation period varied from 5 to 10 days, the larval phase ranged from 16 to 28 days, and the pupal stage ranged from 7 to 10 days (Table 1, Figure 1). On average, female flies survived for 12.5 days (range, 5-20 days) and male flies survived for 27.5 days (range, 20-30 days). 

**TABLE 1:** Development times of stages of *Pressatia choti* under laboratory conditions.

Developmental stage	Days		
	Minimum	Maximum	Average
Eggs	5	10	7.5
First-instar larvae	5	8	6.5
Second-instar larvae	6	10	8
Third/fourth-instar larvae	5	10	7.5
Pupae	7	10	8.5
**Total**	**28**	**48**	**38**

**FIGURE 1:**
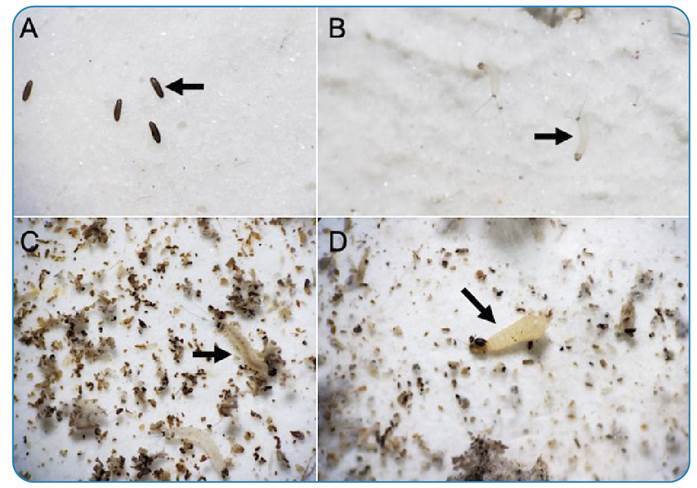
Developmental stages (**A**, egg; **B**, first-instar larva; **C**, third/fourth-instar larva; **D**, pupa) of *Pressatia choti*.

The life cycle of phlebotomine sand flies varies according to the species and environmental conditions. For instance, the life cycle of *Mi. migonei* maintained at the same laboratory conditions (temperature of 26 ºC and 70% relative humidity) ranged from 35 to 45 days and the food for the larvae was a mixture of rabbit feces and rabbit pellets^13^. These conditions are similar to those commonly used for *Lu. longipalpis* rearing^6^.

Another study reported that the average duration of the *Evandromyia lenti* life cycle was ~40 days, under temperature and relative humidity of 26-28 ºC and 80%, respectively^14^. The larval food used in this case was a mix of rabbit feces, vegetal and mineral soil, and dehydrated lettuce in equal proportion plus 2% fish food (Vitormonio)^14^. For *Pintomyia evansi*, the life cycle lasted for ~41 days, at 25 ºC and relative humidity of 89-95%^15^. The food consisted of a 1:1 mixture of rabbit feces and rabbit chow enriched with 5% liver powder^15^. We found no previous studies reporting laboratory colonies of phlebotomine sand flies belonging to the genus *Pressatia* for comparison with the data obtained in the present study.

The establishment of laboratory colonies of insect vectors, such as phlebotomine sand flies is a fundamental step towards development of a range of studies related to the vectors themselves and their interactions with the pathogens they transmit. In conclusion, this study is the first to report the successful establishment and maintenance of *Pr*. *choti* colony, paving the way for future studies on its vector competence and control.
